# Highly efficient eco-friendly corrosion inhibitor for mild steel in 5 M HCl at elevated temperatures: experimental & molecular dynamics study

**DOI:** 10.1038/s41598-019-40149-w

**Published:** 2019-03-06

**Authors:** Muhsen A. M. El-Haddad, A. Bahgat Radwan, Mostafa H. Sliem, Walid M. I. Hassan, Aboubakr M. Abdullah

**Affiliations:** 1 Materials Science & Technology Program, College of Arts and Sciences, Qatar University, Doha, P.O. Box 2713, Qatar; 2 Center for Advanced Materials, Qatar University, Doha, P.O. Box 2713, Qatar; 3 Chemistry Department, Faculty of Science, Cairo University, Giza, 12613 Egypt; 4 Chemistry Department, Faculty of Science, King Abdulaziz University, B.O. 80203, Jeddah, 21589 Saudi Arabia

## Abstract

Laurhydrazide N′-propan-3-one was used as an eco-friendly inhibitor for the corrosion of mild steel in 5 M HCl at elevated temperatures. Various electrochemical techniques and surface characterization methods were utilized in this study. In addition, the kinetics and thermodynamic parameters were calculated and discussed. Furthermore, a geometry optimization of LHP was performed and the time-dependent density functional theory was utilized to calculate the electronic absorption spectra. Finally, frequency calculations were, also, performed on the optimized geometry.

## Introduction

One of the important mineral acids that is widely used in many applications, including well acidizing, water treatment, chemical cleaning, and acid pickling, is hydrochloric acid (HCl)^1–8^. The selection of cost-effective materials to handle this acid requires extreme care and detailed engineering. The presence of certain impurities, such as ferric salts, cupric salts, and chlorine, in the acid and/or a high level of aeration amplifies the oxidizing power of the solution, leading to accelerated corrosion damage^9^. In the petroleum and gas industry, the exposure of materials to acidic environments is more common and frequent than to neutral or alkaline environments^10–13^. This necessitates exploring options and efficient techniques to mitigate and control the corrosion of the different types of steel as they constitute a large fraction of the metallic materials that are exposed to acidic media. Corrosion inhibitors are widely utilized to mitigate corrosion risks. For example, Umoren^14^ investigated the corrosion inhibition of polypropylene glycol for X60 pipeline steel in 15% HCl. The corrosion inhibition efficiency (*IE*%) was found to be 90% at 55 °C using 1000 ppm of polypropylene glycol. Ituen *et al*.^15^ explored the corrosion inhibition of N-acetyl cysteine (NAC) as a base inhibitor compound with different additives of 5-hydroxytryptophan (5-HTP), glutathione, potassium iodide (KI), and polyethylene glycol in 15% HCl solution for different grades of steel (mild steel, J55, and X80) at elevated temperatures. The optimum composition showed a corrosion protection efficiency of 97% at 90 °C on X80 steel. Ansari *et al*.^16^ synthesized two pyrazolone derivatives (PZ-1 and PZ-2) to study their corrosion inhibition in 15% HCl solution for N80 steel. They found that PZ-1, which contains an additional methyl group, showed a better corrosion *IE*% of 93% at 35 °C in comparison to 85.5% for PZ-2. Yadav *et al*.^17^, prepared two carbohydrate compounds namely BIHT and MIHT as green inhibitors for N80 steel in the aforementioned HCl concentration. It was revealed that the chemisorbed inhibitor (BIHT) had the highest *IE*% of 94.8% at 30 °C. Unfortunately, some of the effective corrosion inhibitors used to mitigate corrosion are highly toxic. Increased environmental awareness and the development of regulations have imposed restrictions on the use of such inhibitors. Additionally, the safe disposal of corrosion inhibitors after use or the treatment of contaminated streams is critical and usually defined as a step in all chemical treatment programs, which adds to the corrosion control cost. The environmental and safety concerns related to corrosion inhibition processes have encouraged researchers to explore alternatives that are eco-friendly and offer acceptable inhibition efficiency, especially in acidizing treatment processes, which cost the state of Qatar, in addition to chemical treatment applications, a direct expenditure close to 8 billion USD per year^18^. Therefore, focus on natural and nature-based products is increased to produce so-called “green inhibitors”^19–22^. However, the harsh acidic and high-temperature environments during drilling and stimulation processes in the oil and gas wells limit the full utilization of such green corrosion inhibitors. The development of green corrosion inhibitors that withstand severe acidic corrosive environments and cog the carbon steel corrosion, particularly in well acidizing, is highly desired, especially in the carbonate formations found in the state of Qatar and many other countries.

In this work, laurhydrazide N′-propan-3-one (LHP) is tested as an inexpensive and highly efficient green inhibitor for the corrosion of mild steel (MS) in 16% HCl solution (5 M) at various temperatures using the potentiodynamic polarization (PDP) and electrochemical impedance spectroscopy (EIS) techniques. In addition, several surface characterization methods are used, such as scanning electron microscopy (SEM) coupled with an energy-dispersive X-ray spectroscopy (EDX) unit, atomic force microscopy (AFM), and X-ray photoelectron spectroscopy (XPS). Furthermore, the kinetics and thermodynamic parameters are measured and/or calculated. A geometry optimization of LHP is also performed and the time-dependent density functional theory was used to calculate the electronic absorption spectra. Furthermore, frequency calculations are also performed on the optimized geometry.

## Experimental Work

### Materials and Material Preparation

The elemental analysis of the MS (MS) used in this work, shown in Table 1, was performed using the ARL 3460 optical emission spectrometer (ThermoFisher Scientific, Waltham, MA, USA).

**Table 1:** Elemental composition analysis for MS.

Element	C	Si	Mn	S	P	Cu	Fe
Weight %	0.128	0.25	0.7	0.03	0.04	0.15	Bal.

**Figure 1:**
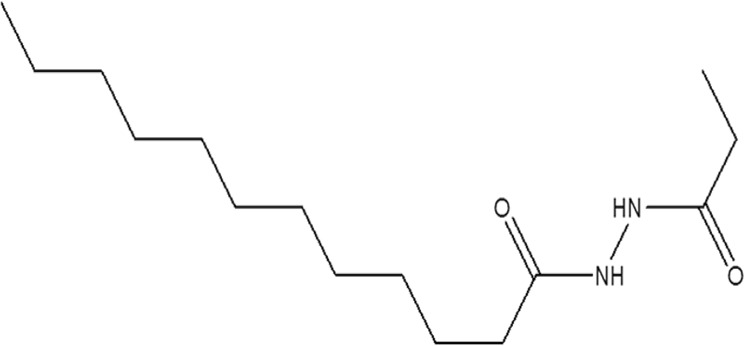
Laurhydrazide N′-propan-3-one chemical structure.

Coupons of equal size (1.5 × 1.5 × 0.5 cm^3^) were cold-cut from a MS plate. They were polished using SiC emery papers from 250 down to 4000 grit, washed with ethanol for 10 min in an ultrasonic bath, then degreased with acetone for 1 min, followed by rinsing with ethanol and finally with deionized (DI) water. Finally, they were dried using air. The corrosive acidic solution that was used in this work was prepared by diluting analytical HCl from 36 to 16% (5 M) using DI water. The chemical structure of LHP (purchased from Shanghai Dejun Technology Co., Ltd, Shanghai, China) is shown in Fig. 1.

### Electrochemical Measurements

A 250 ml double-jacketed three-electrode corrosion cell was used, in which a graphite rod, MS coupon (only 0.5 cm^2^ is exposed to the corrosive solution), and saturated calomel electrode were the counter, working and reference electrodes, respectively. SCE was used with a Luggin capillary to minimize the *IR* drop. The temperature of the cell was controlled 20 and 80 °C using a thermostat water circulator (Julabo F12, Seelbach, Germany). A thermometer was used to monitor the temperature of the electrolyte before and during the experiments. A Reference 300 GAMRY (Warminster, PA, USA) was the potentiostat used to measure the EIS and PDP curves. The GAMRY measurement software packages include EIS300 for EIS and DC105 for corrosion analysis. The free corrosion potential (*E*_OCP_) was stabilized before any electrochemical testing by placing it in the solution for 30 min. EIS was performed within a frequency range of 1 × 10^−1^ to 1 × 10^5^ Hz, with a 5 mV AC peak-to-peak amplitude. PDP curves were measured between −250 and +250 mV with respect to *E*_OCP_ in the more noble direction. The sweep rate was always 0.3 mV s^−1^. Four concentrations of the corrosion inhibitor were used: 92, 185, 277, and 370 μmol L^−1^.

### Surface Analysis

Surface analysis has a vital role in characterizing the surface morphology and studying the effect of the inhibitor and its interaction with the substrate. Immersion tests were conducted to quantify the effect of the corrosive acidic medium on the MS electrode and to study the surface topography before and after the addition of the LHP inhibitor. Three steel coupons were ground and then polished to a mirror-like finish using alumina suspensions of different particle size. The coupons were immersed in the LHP -free 5 M HCl solution for 24 h at 25 °C. The same procedure was repeated, but with the addition of 370 μmol L^−1^ of LHP. The morphology of the two samples was examined and compared using SEM (FEI NOVA NANOSEM 450, Hillsporo, OR, USA), typically operated with an acceleration voltage of 20 kV coupled with an EDX unit. The adsorbed inhibitors on the MS were analyzed using XPS (AXIX Ultra DLD, Kratos, UK), employing a monochromatic Al Kα X-ray source. AFM (MFP-3D, Asylum Research, Goleta, CA, USA) was used in the non-contact tapping mode in air for determining the surface roughness.

### Computational

The geometry optimization was performed using the wb97xd density functional method and 6-311++ g(d, p) as a basis set^23^. The time-dependent density functional theory was utilized to calculate the electronic absorption spectra (using 30 excited states) and the frequency calculations were also performed on the optimized geometry. All these simulations were performed using the Gaussian 09 software package^24^. The electronic absorption spectra were collected using a UV–Vis peak half-width of 0.15 eV. The visualizations were created using the Chemcraft software package^25^. The molecular simulations were created using the adsorption locator tool in Materials Studio^26^. Adsorption Locator identifies possible adsorption configurations by carrying out Monte Carlo searches of the configurational space of the substrate-adsorbate system as the temperature is slowly decreased using simulated annealing. In our calculation we implement the universal force field with fine quality. The surfaces were constructed from pure iron metal followed by a 100, 110, or 111-orientation surface cleavage with a thickness of two layers and a 30 Å vacuum slab. A super cell with dimensions of 10 × 10 was utilized to measure the adsorption energy and the geometry of the adsorbed molecules. The GaussView 5.0 software package was further applied in visualizing the graphical isosurfaces of the electron density.

## Results and Discussion

### Potentiodynamic Polarization Studies

**Figure 2:**
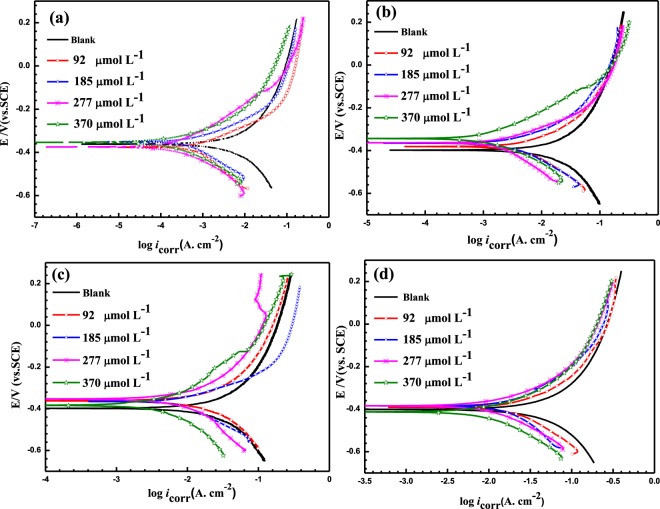
PDP curves of MS in 5 M HCl before and after the addition of different concentrations of LHP at (**a**) 20, (**b**) 40, (**c**) 60, and (**d**) 80 °C.

Figure 2 illustrates the PDP curves at different temperatures for MS in 5 M HCl at various LHP concentrations. The values of *E*_corr_, cathodic (*β*_c_) and anodic (*β*_a_) Tafel slopes besides the corrosion current density (*i*_corr_) are listed in Table 2. In addition, the *IE*% and surface coverage, *θ* in Table 2 are calculated using Equations (1) and (2), respectively^27^.

1 $$IE \% =(\frac{{i}_{1}-{i}_{2}}{{i}_{1}})\times 100$$

2 $$\theta =\frac{IE \% }{100\,}$$

where *i*_1_ and *i*_2_ are the corrosion current densities in the absence and presence of LHP, respectively. Furthermore, the Stern–Geary equation, Equation (3), is utilized to calculate the polarization resistance, R_p_^28^:

3 $${R}_{{\rm{p}}}=\frac{{\beta }_{{\rm{c}}}\,{\beta }_{{\rm{a}}}}{2.303\,{i}_{{\rm{corr}}}({\beta }_{{\rm{c}}}+{\beta }_{{\rm{a}}})}$$

**Table 2:** PDP parameters at various temperatures for MS in 5 M HCl at different concentrations of LHP.

*T* (°C)	*C*_inh_ µmol L^−1^	${\boldsymbol{\beta }}$_a_ (V decade^−1^)	${-{\boldsymbol{\beta }}}_{{\rm{c}}}$ (V decade^−1^)	*R*_*p*_ Ω cm^2^	*E*_corr_ (mV) SCE	*i*_corr_ (mA cm^−2^)	*IE%*	${\boldsymbol{\theta }}$
20	—	0.14	0.19	25.1	358	1.4	—	—
	92	0.1	0.162	51.6	376	0.52	62	0.62
	185	0.08	0.153	76.1	349	0.3	78	0.78
	277	0.06	0.146	97.2	380	0.19	86	0.86
	370	0.03	0.131	117.7	354	0.08	94	0.94
40	—	0.21	0.261	9.7	399	5.2	—	—
	92	0.184	0.241	18.8	380	2.4	53	0.53
	185	0.179	0.221	25.2	362	1.7	67	0.67
	277	0.165	0.211	34.6	358	1.16	77	0.77
	370	0.153	0.209	51.8	342	0.74	86	0.86
60	—	0.23	0.298	4.8	396	11.7	—	—
	92	0.188	0.285	6.9	360	7.1	39	0.39
	185	0.18	0.281	8.2	364	5.8	50	0.5
	277	0.171	0.276	10.7	354	4.3	63	0.63
	370	0.165	0.269	14.3	347	3.1	73	0.73
80	—	0.242	0.302	3.4	398	17.2	—	—
	92	0.197	0.32	3.8	390	13.9	19	0.19
	185	0.185	0.298	4.5	386	11.1	35	0.35
	277	0.181	0.287	5.4	382	8.9	48	0.48
	370	0.173	0.278	7.1	412	6.6	61	0.61

Table 2 reveals the direct relation between the inhibitor concentration and the reduction in the rate of corrosion. It can be noticed that by increasing the inhibitor concentration, the corrosion current density (*i*_corr_), decreases. However, the corrosion current density (*i*_corr_), increases by increasing the temperature. A maximum *IE*% of 94% is attained at 20 °C using 370 µmol L^−1^ of LHP. Elevating the temperature to 80 °C decreases the *IE*% to 61%. The parallel anodic and cathodic Tafel lines and their slopes suggest that the cathodic reaction is primarily activation-controlled, i.e. cathodic and anodic currents were moved to lower values at the same potential with increasing the inhibitor concentration at elevated temperatures. This behavior indicates that both the anodic dissolution and cathodic reactions were suppressed, i.e., LHP is a mixed-type inhibitor. Moreover, an 85 mV shift in *E*_corr_, in the more or less noble directions after the addition of an inhibitor, the inhibitor is classified as an anodic or cathodic inhibitor, respectively^29^. Otherwise, the inhibitor is considered as mixed type category, i.e., it suppresses both reactions^30–32^. That is, the explored compound inhibit the anodic dissolution of the metal surface in the 5 M HCl in addition to the cathodic reaction.

Figure 2 shows shoulders in the anodic curves at high inhibitor concentrations, at all temperatures except for 80 °C. This can be attributed to the alteration of the surface area covered with the corrosion inhibitor, because of the rearrangement of the adsorbed inhibitor molecules on the electrode surface and/or the destabilization or delamination of the thin protective layer formed over the substrate. Additionally, a change in the adsorption or desorption rate of the inhibitor molecules or a local change in the inhibition mechanism for the anodic reaction^33^ could be reasons for the appearance of these shoulders.

### EIS Studies

**Figure 3:**
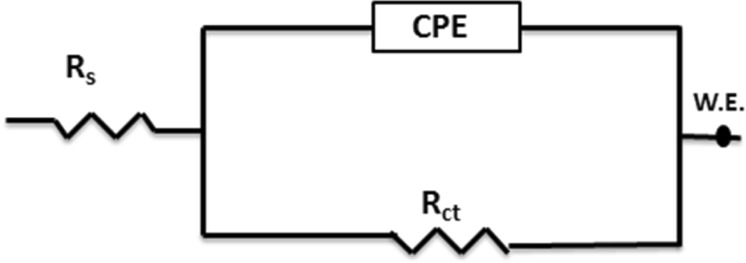
Equivalent circuit used to fit the measured impedance spectra for MS in 5 M HCl.

EIS is widely employed to provide useful information about the kinetics and mechanisms of electrochemical systems^34–37^. Figure 3 shows a typical one time constant equivalent electrical circuit that is used to analyze all the measured EIS data in this study. The used one time constant equivalent circuit indicate that the adsorbed inhibitor forms a monolayer on the MS surface^38^.

It consists of a charge transfer resistance (*R*_ct_), solution resistance (*R*_s_), and constant phase element (CPE) used to describe the non-ideal behavior of the double layer, which is mainly attributed to non-uniform surface coverage and/or surface roughness.

The impedance of the CPE is analyzed using Equation 4^39^:

4 $${Z}_{Q}={[{Y}_{0}{(jw)}^{n}]}^{-1}$$

where *Z*_Q_ represents the CPE impedance (Ω cm^−2^), *Y*_0_ is the CPE constant, *ω* is the frequency in rad s^−1^, and the values of *n* range between 0 and 1 and define the divergence from capacitance linearity. When *n* = 1, *Y*_0_ is equivalent to that of an ideal capacitor. When *n* = 0, *Y*_0_ is equivalent to that of a resistor.

**Figure 4:**
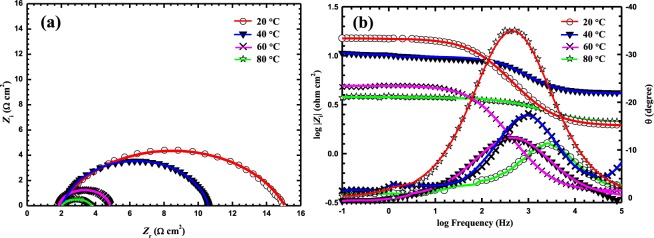
EIS: (**a**) Nyquist plots and (**b**) Bode plots for MS in a LHP – free 5 M HCl at various temperatures.

The measured (dotted lines) and fitted (solid lines) impedance spectra shown in Fig. 4 are for MS in a LHP-free 5 M HCl solution at 20, 40, 60, and 80 °C. It is clear that the semicircles decrease in size as the temperature increases.

**Figure 5:**
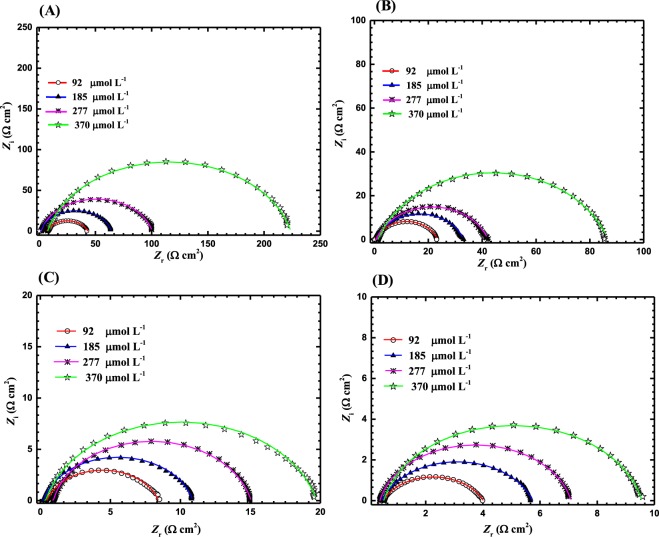
EIS Nyquist plots MS in 5 M HCl with various concentrations of LHP at (**A**) 20, (**B**) 40, (**C**) 60, and (**D**) 80 °C.

**Figure 6:**
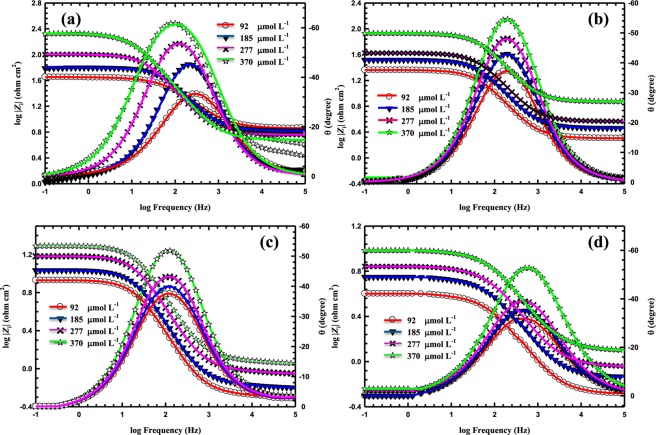
EIS Bode for MS in 5 M HCl in the presence of 92, 185, 277, and 370 µmol L^−1^ of LHP at (**a**) 20, (**b**) 40, (**c**) 60, and (**d**) 80 °C. The symbols are the measured data and the solid lines are the fittings using the equivalent circuit shown in Fig. 3.

Figures 5 and 6 show the Nyquist and Bode plots, respectively, of MS in a 5 M HCl solution with various concentrations of LHP at (b) 20, (c) 40, (d) 60, and (e) 80 °C. It can be noticed from the Nyquist plots that the diameter of the semicircle increases as the corrosion inhibitor concentration increases at different temperatures. The low impedance modulus (*Z*) increases in line with the increase in the LHP concentration due to the increased amount adsorbed of LHP on the metallic substrate. It can be seen from the Bode plots, that the values of phase angle (θ), for the inhibited samples are higher than that of the uninhibited MS at elevated temperature. The increased values of the phase angle for the specimens in the inhibited solutions indicated that the metallic surface significantly becomes smooth due to the formation of protective layer by the adsorbed inhibitors over the MS surface.

Table 3 shows the EIS parameters derived from Figs 4–6 using the equivalent circuit shown in Fig. 3. The *IE*% is calculated using Equation (5)^40^:

5 $$IE \% =(\frac{{R}_{{\rm{ct}}2}-{R}_{{\rm{ct}}1}}{{R}_{{\rm{ct}}1}})\times 100$$

where *R*_ct2_ and *R*_ct1_ represent the charge transfer resistances in the inhibited and uninhibited test solutions, respectively. *θ* is calculated using Equation 2.

**Table 3:** EIS parameters at various temperatures for MS in 5 M HCl with various concentrations of LHP.

*T* (°C)	*C*_inh_, µmol L^−1^	*R*_ct_, Ω cm^2^	*CPE*		*C*_dl_, µF	*IE*%	$\theta$	Goodness of fit (χ^2^)
			*Y*_0_ × 10^−6^ *s*^*n*^ Ω ^−1^ cm^−2^	*n*				
20	0	15	751	0.810	258	—	—	6.7 × 10^−5^
	92	44	641	0.743	186	65.8	0.66	5.6 × 10^−4^
	185	62	610	0.720	171	75.8	0.76	1.3 × 10^−4^
	277	100	550	0.697	162	84.9	0.85	2. 2 × 10^−5^
	370	210	524	0.625	140	92.8	0.93	3.2 × 10^−4^
40	0	11	772	0.826	280	—	—	3.7 × 10^−4^
	92	23	656	0.784	206	54.3	0.54	4.8 × 10^−4^
	185	33	621	0.766	189	68.2	0.68	7.2 × 10^−5^
	277	42	566	0.758	171	75	0.75	4.4 × 10^−5^
	370	85	533	0.734	148	87.6	0.87	6.1 × 10^−4^
60	0	5	818	0.857	324	—	—	8.4 × 10^−5^
	92	9	781	0.827	273	43.5	0.44	5.3 × 10^−4^
	185	11	761	0.801	230	55.6	0.56	7.5 × 10^−4^
	277	15	732	0.789	219	68.4	0.68	9.3 × 10^−5^
	370	20	714	0.761	186	75.4	0.75	2.8 × 10^−4^
80	0	3	885	0.894	444	—	—	1.2 × 10^−5^
	92	4	826	0.864	336	15	0.15	6.7 × 10^−4^
	185	6	806	0.839	284	38.1	0.38	9.5 × 10^−4^
	277	7	784	0.816	242	51.4	0.51	1.8 × 10^−4^
	370	10	764	0.792	210	64.6	0.65	4.9 × 10^−4^

*R*_ct_, *Y*_0_, and *n* are used to calculate the metal solution interface double layer capacitance (*C*_dl_) using Equation (6)^41^:

6 $${C}_{{\rm{dl}}}={({Y}_{0}{{R}_{ct}}^{1-n})}^{1/n}$$

Table 3 reveals that *R*_ct_ increases as LHP concentration is raised. This is attributed to the adsorbed molecules of LHP on the MS forming a barrier film, which restricts the accessibility of Cl^−^ ions to the metal surface. On the contrary, the double layer capacitance (*C*_dl_) gradually decreases as the inhibitor concentration increases. The decrease in the *C*_dl_ is primarily caused by (i) the increase in the thickness of the double layer thickness (δ) because of the adsorption of the LHP onto the MS surface and/or (ii) the decrease in the dielectric constant (*ε*) due to the replacement of water molecules by the LHP ones as indicated by the Helmholtz equation^42^:

7 $${C}_{{\rm{dl}}}=\frac{{\rm{\varepsilon }}\,{{\rm{\varepsilon }}}_{0}\,A}{\delta }$$

where *A* represents the cross-sectional area of the electrode, and *ε*_0_ and *ε* represent the dielectric constants of air and water, respectively.

As the *IE%* is directly proportional to *R*_ct2_, it also increases as the LHP concentration increases and decreases as the temperature is raised, as seen in Table 3.

It is worth noting that *n* has the highest values in LHP-free solution at any temperature, and decreases as the LHP dosage increases at a given temperature. In addition, the higher the temperature is, the higher is the value of *n* at any concentration of LHP. The values of *n* (approaching unity), indicate that the *CPE* is getting closer to the ideal capacitor behavior. It is noteworthy that the EIS parameters summarized in Table 3, are consistent with the Tafel analyses shown in Table 2.

### Adsorption Isotherm and Thermodynamic Calculations

Adsorption isotherms are used extensively to illustrate and characterize the interaction between the applied corrosion inhibitor and a metallic substrate.

**Figure 7:**
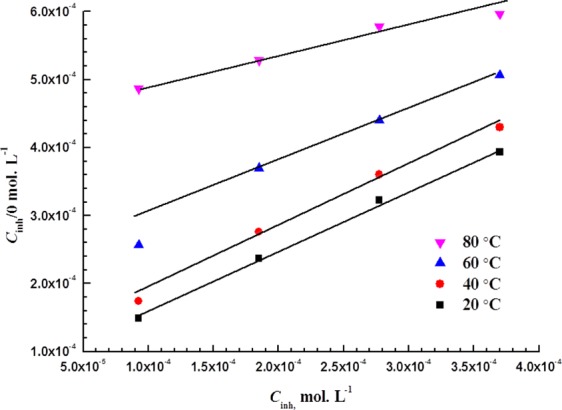
Langmuir adsorption plots at various temperatures for MS in 5 M HCl.

Langmuir is found to be the best isotherm by far that fits the measured experimental data. The Langmuir isotherm relates *C*_inh_ and *θ* as follows^43^:

8 $$\frac{{C}_{{\rm{inh}}}}{\theta }=\frac{1}{{K}_{{\rm{ads}}}}+{C}_{{\rm{inh}}}$$

where *C*_inh_ is the LHP concentration and *K*_ads_ is the adsorption equilibrium constant, which can be obtained from the intercept of the plots shown in Fig. 7.

Figure 7 shows the relation between *C*_inh_/*θ* and *C*_inh_ at various temperatures. The standard Gibbs free energy change of adsorption (${\rm{\Delta }}{G}_{{\rm{ads}}}^{o}$) can be readily obtained using Equation (9) after getting the constants of adsorption (*K*_ads_) at different temperatures from the (*C*_inh_/*θ*)-intercepts of the plots in Fig. 7.

9 $${K}_{{\rm{ads}}}=\frac{1}{55.5}{e}^{-\frac{{\rm{\Delta }}{G}_{{\rm{ads}}}^{{\rm{o}}}}{RT}}$$

where *R* equals 8.314 j mol^−1^ K^−1^, *T* is the temperature, and the 55.5 is the number of moles of water in 1 liter^20^.

**Figure 8:**
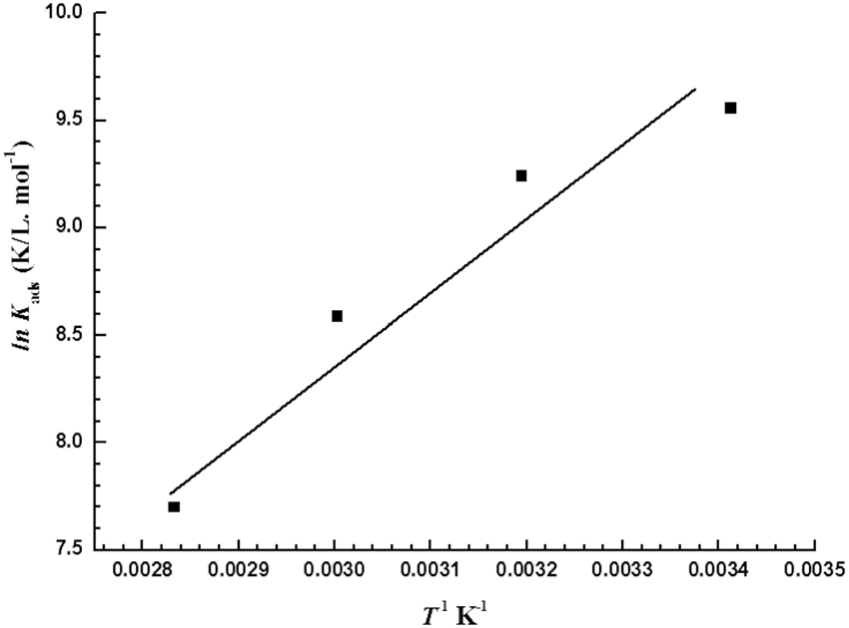
ln *K*_ads_ vs. *T*^−1^ for the LHP at the MS surface in 5 M HCl.

By plotting ln *K*_ads_ versus *T*^−1^, as shown in Fig. 8, a straight line is obtained, which follows the van’t Hoff equation^19^:

10 $$\mathrm{ln}\,{K}_{{\rm{ads}}}=\frac{-{\rm{\Delta }}{{H}^{0}}_{{\rm{ads}}}}{RT}+\frac{{\rm{\Delta }}{{S}^{0}}_{{\rm{ads}}}}{R}$$

where ${\rm{\Delta }}{S}_{{\rm{ads}}}^{0}$ and ${\rm{\Delta }}{H}_{{\rm{ads}}}^{0}$ are change in the entropy and standard enthalpy of adsorption, respectively.

By determining ${\rm{\Delta }}{H}_{{\rm{ads}}}^{0}$ from Equation (10), ${\rm{\Delta }}{S}_{{\rm{ads}}}^{0}$ is calculated utilizing either Equation (10)^44^ or Equation (11).

11 $${\rm{\Delta }}{G}_{{\rm{ads}}}^{0}={\rm{\Delta }}{H}_{{\rm{ads}}}^{0}\,-\,T{\rm{\Delta }}{S}_{{\rm{ads}}}^{0}$$

Table 4 lists the ${\rm{\Delta }}{H}_{{\rm{ads}}}^{0}$, ${\rm{\Delta }}{S}_{{\rm{ads}}}^{0}$, *K*_ads_, and ${\rm{\Delta }}{G}_{{\rm{ads}}}^{o}$ values for the adsorption of LHP at the MS surface in 5 M HCl.

**Table 4:** Thermodynamic parameters derived from Figs 7 and 8.

Temperature, K	*K*_ads_, L mole^−1^	${\boldsymbol{\Delta }}{{\boldsymbol{G}}}_{{\bf{ads}}}^{{\bf{0}}}$_,_ kJ mol^−1^	${\boldsymbol{\Delta }}{{\boldsymbol{H}}}_{{\bf{ads}}}^{{\bf{0}}}$, kJ mol^−1^	${\boldsymbol{\Delta }}{{\boldsymbol{S}}}_{{\bf{ads}}}^{{\bf{0}}}$_,_ J mol^−1^ K^−1^
293.15	14139	−33.1	−27.7	18.5
313.15	10337	−34.5	−27.7	21.8
333.15	5354	−34.8	−27.7	21.4
353.15	2211	−34.4	−27.7	19.1

The positive values of ${\rm{\Delta }}{S}_{{\rm{ads}}}^{0}$ indicate an increase in the entropy due to the adsorption of LHP on the MS surface. This is attributed to the exothermic nature of the adsorption process, as seen from the negative values of ${\rm{\Delta }}{H}_{{\rm{ads}}}^{0}$. The high values for the constant of adsorption (*K*_ads_), particularly at 25 °C, for the studied LHP corrosion inhibitor indicate strong adsorption on the MS substrate. Large *K*_ads_ values suggest a strong adsorption tendency and hence a better inhibition performance. This is explained by the presence of π-electrons in the inhibitor’s inherent molecular structure.

The negative values of ${\rm{\Delta }}{G}_{{\rm{ads}}}^{0}$ are aligned with the spontaneity of the LHP adsorption on the MS surface (as -R*T* ln *K*_ads_ has negative values too). The values of ${\rm{\Delta }}{G}_{{\rm{ads}}}^{0}$ are usually interpreted in relation to the nature of the adsorption process: whether it is physisorption or chemisorption. Generally, if the values of ${\rm{\Delta }}{G}_{{\rm{ads}}}^{0}$ are more than −20 kJ mol^−1^, then the physisorption mechanism is favored, whereas if the values of ${\rm{\Delta }}{G}_{{\rm{ads}}}^{0}$ are −40 kJ mol^−1^ or lower, then the adsorption process is chemisorption.

As seen in Table 4, the obtained ${\rm{\Delta }}{G}_{{\rm{ads}}}^{0}$ ranges from −33.1 to −34.8 kJ mol^−1^, which is between −20 and −40 kJ mol^−1^. Thus, the process cannot be classified as chemisorption or physisorption. Rather, it is a mix of chemisorption and physisorption.

### Effect of Activation Energy and Temperature on the Corrosion Rate

The inhibition mechanism and efficiency are directly influenced by the activation energy (*E*_a_). The rate of most chemical reactions tends to increase as the temperature increases. The effect of temperature on the corrosion rate of MS can be evaluated using the Arrhenius equation:

12 $$\mathrm{log}\,CR=\,\mathrm{log}\,A-\frac{\,{E}_{{\rm{a}}}}{2.303\,RT}$$

where *CR* is the corrosion rate expressed in terms of *i*_corr_ at a specific temperature (*T*), (*E*_a_) is the activation energy, *R* = 8.314 J mol^−1^ K^−1^, and *A* is the Arrhenius constant, which is affected by the metal type and electrolyte composition.

**Figure 9:**
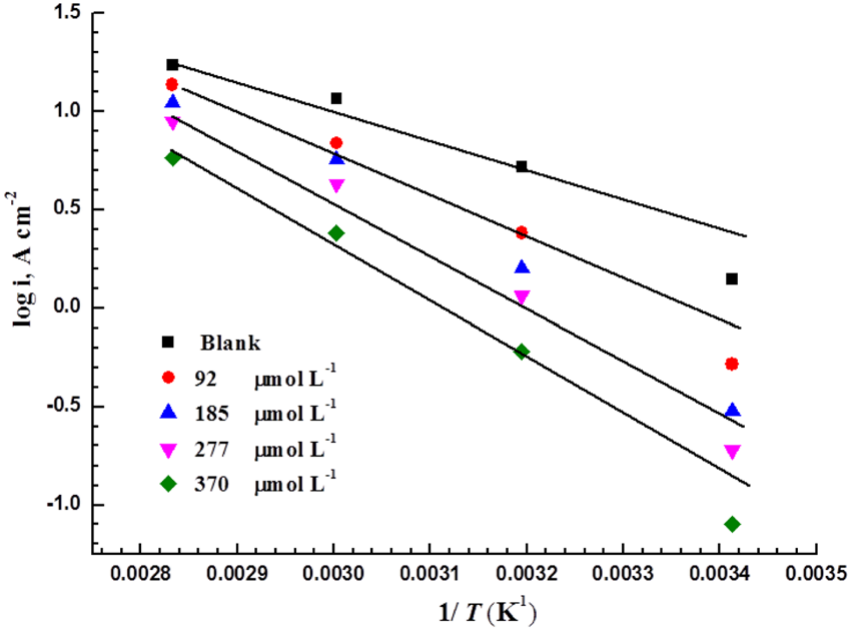
Arrhenius plots for MS in 5 M HCl with various concentrations of LHP.

The Arrhenius plots of the MS before and after the addition of the corrosion inhibitor are given in Fig. 9, with a linear regression rate that is close to unity.

The calculated *E*_a_ are listed in Table 5. *E*_a_ increases as the inhibitor concentration is raised. The higher value of *E*_a_ is referred to the formed barrier film of LHP molecules at the MS surface.

**Table 5:** Activation energy (*E*_a_), regression coefficient (*r*^2^), ∆H*, and ∆S* for MS in 5 M HCl with various concentrations of LHP.

Conc. of inhibitor (µmol L^−1^)	*E*_a_ (kJ mol^−1^)	*r* ^2^	ΔH^*^ (kJ mol^−1^)	ΔS^*^ (J mol^−1^ K^−1^)
0	36.3	0.95	33.6	−126
92	47.1	0.97	44.4	−97
185	52.5	0.97	49.8	−84
277	55.7	0.97	53.1	−76
370	61.7	0.98	61.7	−6

The entropy of activation (∆S*) and the enthalpy of activation (∆H*) due to the dissolution of MS in 5 M HCl are calculated using the Arrhenius equation^33^:

13 $$CR=\frac{RT}{Nh}{\exp }^{\frac{{\rm{\Delta }}{S}^{\ast }}{R}}{\exp }^{\frac{-{\rm{\Delta }}{H}^{\ast }}{RT}}$$

where *h* is Planck’s constant and *N* is Avogadro’s number.

**Figure 10:**
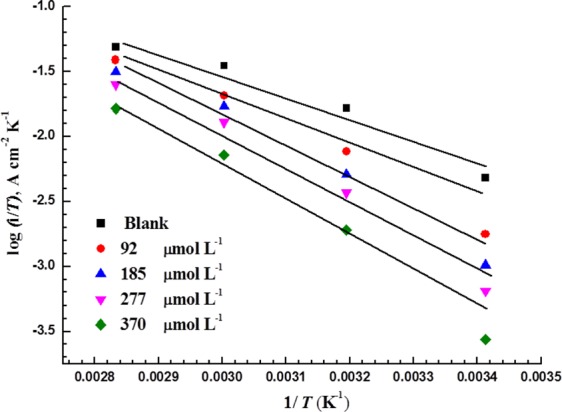
Transition-state plots for MS in 5 M HCl with various concentrations of LHP.

From Fig. 10, ∆H* and ∆S* are obtained from the slope of (∆H*/2.303*R*) and the intercept of [log (*R*/*Nh*) + (∆S*/2.303*R*)] are used to calculate respectively. The average difference between *E*_a_ and ∆H* is approximately 2.6 kJ mol^−1^ for each test, which is almost the value of *RT* (2.63 kJ mol^−1^). This indicates that the dissolution of MS in this environment is a unimolecular reaction.

The thermodynamic parameters of LHP inhibitor were compared with some other reported inhibitors in 5 M HCl as shown in Table 6. It is worth to mention that the reported inhibitors herein, exhibited a mixed type of adsorption.

**Table 6:** Comparison of the energetic parameters of the investigated inhibitor (LHP), and some other reported inhibitors in 5 M HCl.

Inhibitor	ΔH^*^ (kJ mol^−1^)	ΔS^*^ (J mol^−1^ K^−1^)	${\boldsymbol{\Delta }}{{\boldsymbol{G}}}_{{\bf{ads}}}^{{\bf{0}}}$_,_ kJ mol^−1^	Type of adsorption	Ref.
Polypropylene glycol	57.7	−120	−29.2	Mixed type	^14^
Propargyl alcohol	21.7	21.8	−25.7	Mixed type	^54^
5-(4-methoxyphenyl)-3-(4-methylphenyl)4,5-dihydro-1H-pyrazol-1-yl-(pyridin-4-yl)methanone	−64.3	−88	−39.7	Mixed type	^55^
1-diphenylaminomethyl-3-(1-N-dithiooxamide) iminoisatin	−58.7	49.4	−38.2	Mixed type	^56^
Xanthan gum-graft-poly(acrylamide)	98.8	92.3	−24.01	Mixed type	^57^

### Surface Topography and Characterization

#### SEM analysis

**Figure 11:**
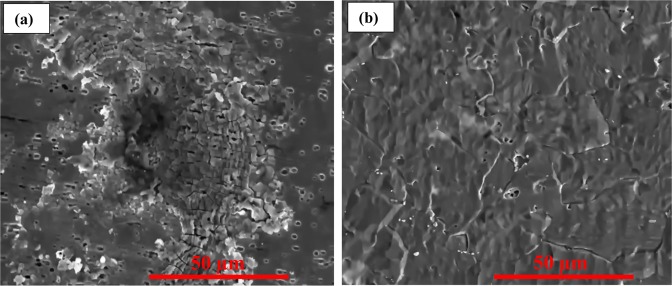
SEM surface analysis micrograph for (**a**) MS immersed in 5 M HCl and (**b**) after the addition of 370 µmol L^−1^ of LHP at 20 °C for 24 h.

Figure 11 depicts SEM micrographs that show the morphology of the MS surface after immersion for 24 h in (a) a LHP -free 5 M HCl and (b) 5 M HCl with 370 µmol L^−1^ of LHP at 20 °C. Figure 11a shows that the examined surface is heavily corroded. However, for the immersion in the presence of 370 µmol L^−1^ of LHP, the corrosion looks more uniform.

EDX is conducted to examine the presence of nitrogen on the corroded surfaces in HCl with 370 µmol L^−1^ of the LHP at 20 °C. The presence of nitrogen on the metallic substrate is confirmed to be 1.9%, which demonstrates the adsorption of LHP on the MS surface.

#### XPS analysis

**Figure 12:**
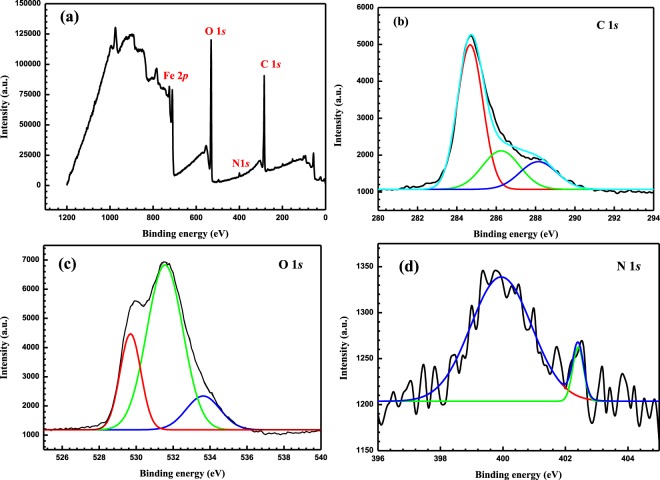
(**a**) XPS survey scan composition of the MS immersed in 5 M HCl with 370 µmol L^−1^ of LHP at 20 °C for 24 h and the profiles of (**b**) C 1 *s*, (**c**) O 1 *s*, and N 1 *s*.

The XPS survey of the adsorbed inhibitor and the high-resolution XPS analysis of C, O, and N are shown in Fig. 12 for MS after immersion in 5 M HCl with 370 µmol L^−1^ of LHP O at 20 °C. The C 1 *s* is deconvoluted into three different peaks. A peak at 284.7 eV is related to the C-C of the adsorbed inhibitor^45^. In addition, a second peak at 288.1 eV is credited to the existence of -C=O groups^46,47^ and a third one at 286.2 eV for the C-N of the adsorbed LHP on the protected MS surface^48^. The deconvolution of the O 1 *s* spectrum yields three peaks. The first at 529.6 eV is related to O^2−^, which is mainly is associated with oxygen atoms bonded to Fe_2_O_3_^49^. The second one at 531.5 eV is attributed to the OH^−^ of FeOOH^50^. The third peak observed at 533.5 eV could be ascribed to the existence of oxygen in the adsorbed water^4^. On the contrary, the deconvolution of the N 1 *s* spectrum peak results in two peaks around 399.8 and 402.3 eV, which are mainly referred to the N atoms bonded to the MS surface (N-Fe) and to the protonated nitrogen atoms of the hydrazine group, respectively^17^. XPS confirms the presence of the adsorbed LHP corrosion inhibitor on the metal surface.

#### Atomic force microscopy analysis

**Figure 13:**
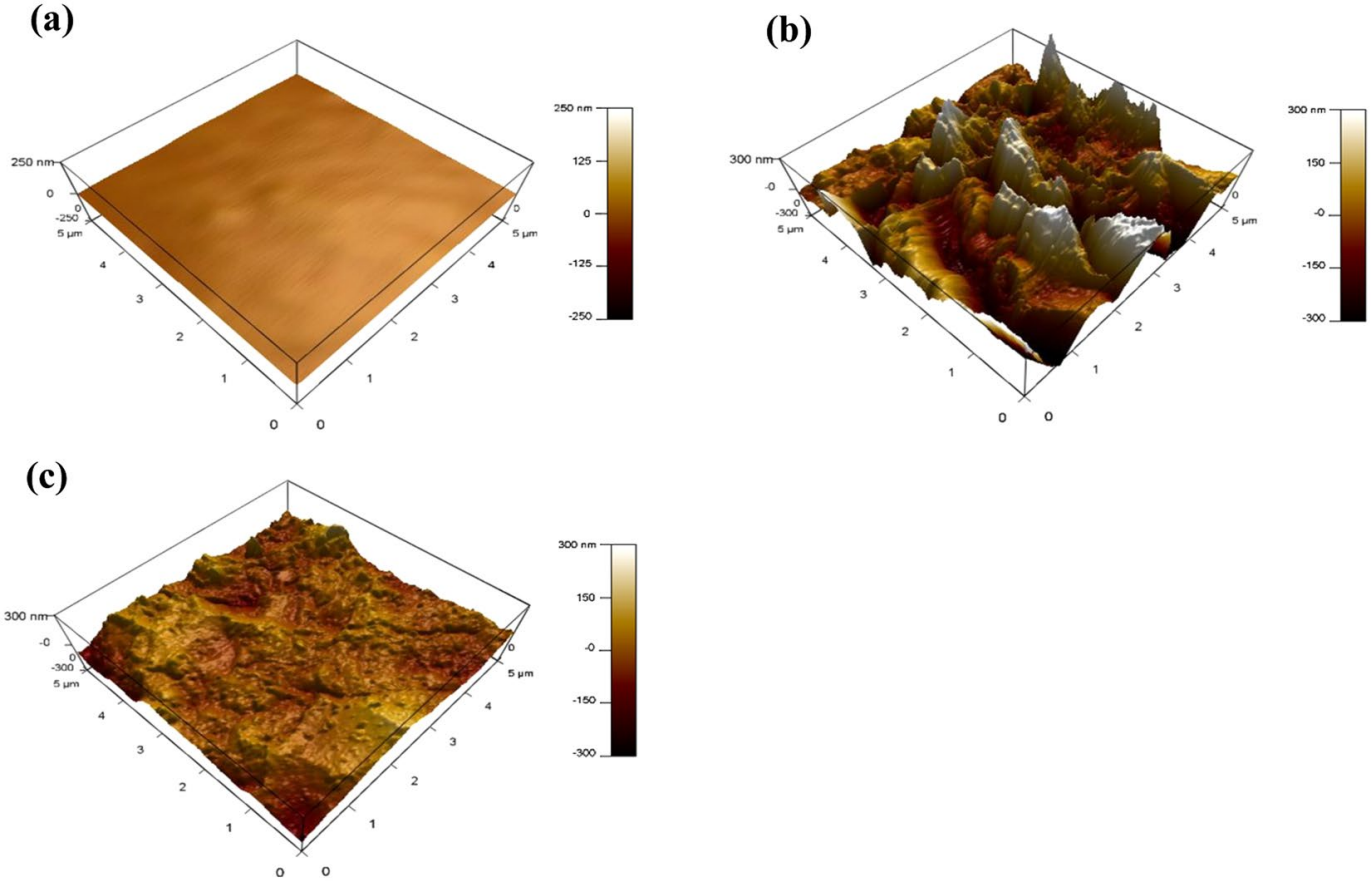
AFM images for (**a**) the polished MS surface, (**b**) after immersion in 5 M HCl and (**c**) after the addition of LHP for 24 h at 20 °C.

AFM is a powerful technique that has been widely used to explore the effect of inhibitor on the surface roughness and topography of the metal surface in aggressive media^33,51–53^. Figure 13 shows three-dimensional images of two MS samples that had been ground using SiC to 4000 grit and immersed in 5 M HCl for 24 h with and without 370 µmol L^−1^ of LHP. The average roughness of a MS surface in the absence and presence of LHP in 5 M HCl is approximately 43.4 and 16.4 nm, respectively. The appreciable decrease in the surface roughness after the addition of LHP demonstrates the good inhibition performance of LHP in 5 M HCl.

### Structural Geometrical Parameters of the Optimized Molecule

**Figure 14:**
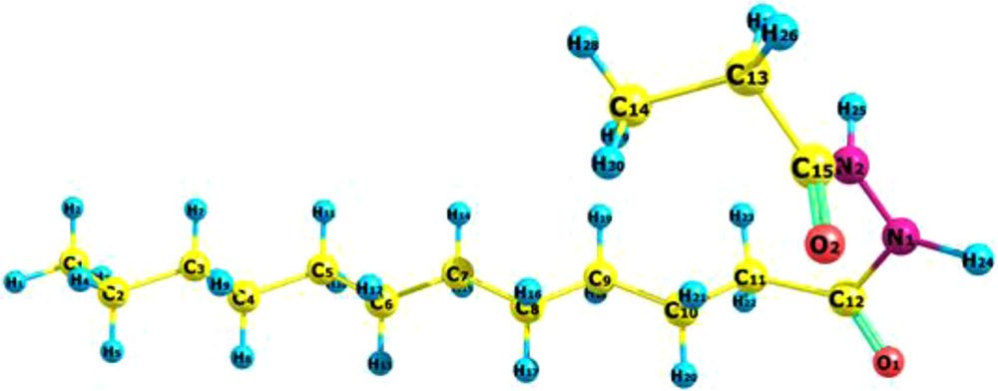
Optimized geometry of LHP using the wb97xd/6-311++ g(d, p) level of the calculations.

The optimized geometry of the studied LHP results in the structure shown in Fig. 14. The optimized geometry shows no imaginary frequencies, which suggests the stability of the converged structure.

The asymmetric and symmetric vibrational bands of C=O are located at 1812 and 1827 cm^−1^, respectively. The vibrational stretching bands of the two N-H groups are located at 3604 and 3658 cm^−1^, respectively. The aliphatic straight chain with 10 C atoms has similar C-C bonds with an average value of 1.526 Å for the C-C bonds between C1 and C10, whereas the C10-C11, C11-C12, C13-C14, and C13-C15 bond lengths are 1.536, 1.511, 1.532, and 1.514 Å, respectively. Both C15=O and C12=O have the same bond length of 1.21 Å. The C15-N2 bond length is slightly shorter than the C12-N1 bond length, by approximately 0.015 Å, with values of 1.376 and 1.391 Å, respectively. The N1-N2 bond length is 1.376 Å. The bond angles C11-C12-O1, O1-C12-N1, N1-C12-C11, C13-C15-O2, O2-C15-N2, and N2-C15-C13 are 123.7°, 119.3°, 116.8°, 123.3°, 122.1°, and 114.6°, respectively, suggesting the sp2 hybridizations whereby C=O bonds take up more space and consequently slightly decrease the N-C-C bond angle. The dihedral angles O2-C15-N2-N1 and O1-C12-N1-N2 are −1.44° and −165.35°, suggesting more deviation than planarity on the side of the longer aliphatic chain.

The natural bond order charge analysis showed that the most negative atoms are O1 and O2 with values of −0.606 and −0.618, followed by N1 and N2 with values of −0.481 and −0.472, respectively. The most positive atoms are C12 and C13, with charges of 0.712 and 0.686, respectively. All the other C atoms in CH_2_ have a partial negative charge of approximately −0.4 that is neutralized by a positive charge of approximately 0.2 on each H atom. The terminal C in CH_3_ carries a larger partial negative charge of approximately −0.577, because it is surrounded by three H atoms each, which have a positive charge of approximately 0.19. The nonlocalized negative charges suggest that more than one interaction site with the metal surface is involved, as opposed to the possibility of a single active site in the adsorption process. The total dipole moment is 5.7294 D, directed approximately above the C9 atom and perpendicular to the C8-C9 bond, which further suggests the non-localized distribution of atomic charges.

#### Molecular simulation

**Figure 15:**
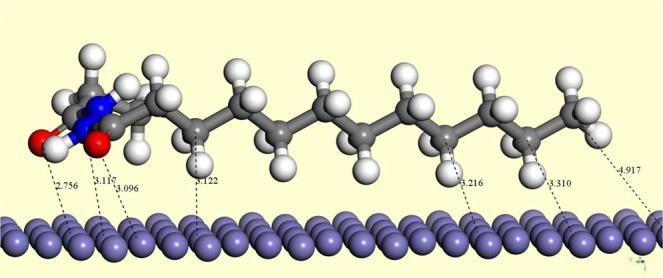
Adsorption of LHP on the 100 Fe surface; bond lengths are in Å.

The molecular simulation of LHP on different pure iron surfaces showed approximately identical adsorbed molecular geometries to that shown in Fig. 15. The whole molecule is adsorbed on the surface, with bond lengths of 4.9, 3.2, 2.9, and 3.1 Å for methyl, CH_2_, O, and N, respectively, as measured relative to the nearest Fe atom on the surface. The molecular simulation suggested that adsorption sites are not localized on one atom, and that the part with the heteroatoms are much more strongly adsorbed than aliphatic hydrocarbon. Upon comparing the geometry of the isolated LHP with the adsorbed one, the main geometrical changes are localized in C15-N2, C12-N1 and N1-N2 bond lengths with 1.359, 1.374, and 1.412 Å, respectively. Both C15-N2 and C12-N1 have shortened by 0.017 Å, which results in elongation of N1-N2 by 0.035 Å. Furthermore, the calculation showed rigid adsorption energies of −2.914, −2.635, and −1.907 kcal mol^−1^ for iron surfaces with orientations of 100, 110, and 111, respectively. Although the values of the adsorption energy are not very different, the adsorption energy values may lead to the conclusion that the 100-iron surface will have the best coverage of the three surfaces, and that the 111 surface will have the weakest coverage.

## Summary and Conclusion

The LHP eco-friendly corrosion inhibitor has shown extraordinary performance at normal and elevated temperatures in inhibiting the corrosion of MS in 5 M HCl. The efficiency was found to increase as the inhibitor concentration increased. When the testing temperature increased to 80 °C, a slight decline in efficiency was noted, due to the increased rate of iron dissolution in the acidic brine and the expected desorption of the attached inhibitor from the metallic substrate. An inhibition efficiency of more than 94% was successfully achieved upon the addition of the candidate inhibitor to a concentration of 370 µmol L^−1^. The relationship between the surface coverage and inhibitor concentration is consistent with Langmuir’s adsorption isotherm. LHP is found to be a mixed type inhibitor. The adsorption of the inhibitor on the steel surface was proven to be chemi–physisorption, which is confirmed by the calculated standard Gibbs free energy (${\rm{\Delta }}{G}_{{\rm{ads}}}^{0}$). The optimized geometry shows no imaginary frequencies, which suggests the stability of the converged structure. In addition, the molecular simulation of LHP proved that the 100-iron surface will have better coverage compared to the 110 surface, while the 111 surface will have the weakest coverage. AFM analyses revealed a significant decrease in the surface roughness under an inhibited condition compared to immersion with no inhibitor added. Additionally, EDS and XPS revealed the presence of nitrogen on the MS, which validates the adsorption of LHP onto the surface. Applications of this inhibitor for the mitigation of acidic corrosion in the petroleum and gas industry will add value in terms of enabling safer operations and contributing to significant cost savings. Further qualification testing of the candidate inhibitor under dynamic conditions is recommended to achieve greater assurance and gain more confidence in the inhibition capabilities.

## Data Availability

The raw data required to reproduce these findings can be shared at any time based on direct requests to the authors.
